# On the estimation of inbreeding depression using different measures of inbreeding from molecular markers

**DOI:** 10.1111/eva.13126

**Published:** 2020-10-23

**Authors:** Armando Caballero, Beatriz Villanueva, Tom Druet

**Affiliations:** ^1^ Centro de Investigación Mariña, Departamento de Bioquímica, Genética e Inmunología, Edificio CC Experimentais Universidade de Vigo Vigo Spain; ^2^ Departamento de Mejora Genética Instituto Nacional de Investigación y Tecnología Agraria y Alimentaria Madrid Spain; ^3^ Unit of Animal Genomics GIGA‐R & Faculty of Veterinary Medicine University of Liège Liège Belgium

**Keywords:** coancestry, deleterious recessive mutations, genetic drift, identity by descent, inbreeding load

## Abstract

The inbreeding coefficient (*F*) of individuals can be estimated from molecular marker data, such as SNPs, using measures of homozygosity of individual markers or runs of homozygosity (ROH) across the genome. These different measures of *F* can then be used to estimate the rate of inbreeding depression (ID) for quantitative traits. Some recent simulation studies have investigated the accuracy of this estimation with contradictory results. Whereas some studies suggest that estimates of inbreeding from ROH account more accurately for ID, others suggest that inbreeding measures from SNP‐by‐SNP homozygosity giving a large weight to rare alleles are more accurate. Here, we try to give more light on this issue by carrying out a set of computer simulations considering a range of population genetic parameters and population sizes. Our results show that the previous studies are indeed not contradictory. In populations with low effective size, where relationships are more tight and selection is relatively less intense, *F* measures based on ROH provide very accurate estimates of ID whereas SNP‐by‐SNP‐based *F* measures with high weight to rare alleles can show substantial upwardly biased estimates of ID. However, in populations of large effective size, with more intense selection and trait allele frequencies expected to be low if they are deleterious for fitness because of purifying selection, average estimates of ID from SNP‐by‐SNP‐based *F* values become unbiased or slightly downwardly biased and those from ROH‐based *F* values become slightly downwardly biased. The noise attached to all these estimates, nevertheless, can be very high in large‐sized populations. We also investigate the relationship between the different *F* measures and the homozygous mutation load, which has been suggested as a proxy of inbreeding depression.

## 1 INTRODUCTION

Inbreeding depression, the change in the mean of a quantitative trait with inbreeding (a reduction in fitness in the case of life‐history traits), is a main factor for the extinction of endangered species (Allendorf, Luikart, & Aitken, 2013; Frankham, Ballou, & Briscoe, 2010; O’Grady et al., 2006) and the management of livestock (Leroy, 2014). Regarding fitness components, inbreeding depression is generally assumed to occur because of the increased homozygosity of partially recessive deleterious alleles (Charlesworth & Willis, 2009; Hedrick, 2012), although some unknown depression may also occur by reduced heterozygosity at loci presenting heterozygote advantage (Charlesworth, 2015).

For quantitative traits, the rate of inbreeding depression (ID) is usually quantified by the slope of the linear regression of the individual phenotypic values on their inbreeding coefficient, *F* (Lynch & Walsh, 1998). The inbreeding coefficient of an individual, the probability of identity by descent of alleles, that is, alleles of an individual in a locus that come from a common ancestor to its parents, can be obtained from pedigree data (Wright, 1969) but also inferred from genome homozygosity (Li & Horvitz, 1953; Malécot, 1948; Toro et al., 2002), mainly using highly dense molecular markers, such as SNPs. Genomic or molecular measures of *F* have the advantage of providing estimates of realized inbreeding values, which are often more precise than pedigree ones (Curik, Ferenčaković, & Sölkner, 2014; Keller, Visscher, & Goddard, 2011; Wang, 2016), and do not require a knowledge of the genealogical relationships of individuals. They, however, measure overall homozygosity, which includes not only identity by descent of alleles but also identity in state, that is, identical alleles coming from different ancestors.

Different metrics are available to obtain the estimates of genomic inbreeding, either based on maximum likelihood (e.g., Milligan, 2003; Wang, 2007), methods of moments (e.g., Purcell et al., 2007; Ritland, 1996), the diagonal elements of a genomic relationship matrix (VanRaden, 2008; Yang, Lee, Goddard, & Visscher, 2011), homozygosity measures (e.g., Bjelland, Weigel, Vukasinovic, & Nkrumah, 2013; Szulkin, Bierne, & David, 2010), genotypic correlations (Ackerman et al., 2017) or the proportion of the genome within runs of homozygosity (ROH) (Ceballos, Joshi, Clark, Ramsay, & Wilson, 2018; Ferenčaković et al., 2013; Ferenčaković, Sölkner, Kapš, & Curik, 2017; McQuillan et al., 2008) or homozygosity‐by‐descent genomic segments (Druet & Gautier, 2017). Multiple empirical comparisons have been made between the estimates of ID with alternative inbreeding measures (Bjelland et al., 2013; Goudet, Kay, & Weir, 2018; Kardos, Taylor, Ellegren, Luikart, & Allendorf, 2016; Pryce, Haile‐Mariam, Goddard, & Hayes, 2014; Santure et al., 2010; Saura et al., 2015; Zhang, Calus, Guldbrandtsen, Lund, & Sahana, 2015), but without the knowledge of the true ID, it is difficult to make conclusions about which genomic *F* measure provides more appropriate estimations.

An assessment of the accuracy of some of the *F* measures in the estimation of ID can be made with computer simulations (e.g., Kardos, Luikart, & Allendorf, 2015; Keller et al., 2011), where the true parameters are known, even though simulated scenarios are always a simplified view of the natural processes. Thus, Keller et al. (2011) studied the correlation between different *F* measures and the homozygous mutation load (HML) of each individual, defined as the number of homozygous loci for rare alleles (MAF < 0.05) carried by an individual. This is assumed to be a proxy of the individual overall load of homozygous (partially) recessive deleterious mutations and, thus, of inbreeding depression. Using this approach, Keller et al. (2011) showed that ROH‐based *F* measures are the most powerful to detect ID. Kardos et al. (2015) rather considered the correlation between the *F* measures and the proportion of the genome which is identical by descent (IBD), concluding that both ROH‐based and SNP‐by‐SNP‐based *F* measures can explain a large amount of the genomic IBD variation if the number of SNPs is sufficiently large (tens of thousands). However, these previous simulation studies did not consider ID of a quantitative trait in itself, but addressed the issue in an indirect way by considering some proxies of it.

More recently, Yengo et al. (2017) used data on true genotypes for several millions of human SNPs and simulated ID by ascribing phenotypic effects to a sample of the SNPs under different scenarios. They then investigated the accuracy in the estimation of ID using different measures of individual *F*. The main conclusion was that the most accurate estimations were obtained with a measure of genomic inbreeding based on the correlation between uniting gametes (*F_UNI_*; previously called *F^III^* by Yang et al., 2011), a statistic based on the deviation of SNP homozygosity from their expected values giving a larger weight to rare alleles than to common alleles. In contrast, ROH‐based measures of inbreeding (*F_ROH_*) were shown to provide very large overestimations of ID. Yengo et al. (2017) thus explicitly recommended the use of *F^III^* to estimate ID with molecular data. This generated some debate, though, as it was argued that the simulations performed by Yengo et al. (2017) were not carried out with explicit simulated individuals subjected to genetic processes (Kardos, Nietlisbach, & Hedrick, 2018). Rather, trait values had been simulated as a function of the inbreeding coefficient which was calculated in a similar way as *F^III^*, perhaps biasing the conclusions in favour of this *F* measure (Kardos et al., 2018; see also reply by Yengo et al., 2018). More recently, Nietlisbach, Muff, Reid, Whitlock, and Keller (2019) performed a set of genetically explicit simulations concluding that, in contrast to Yengo et al. (2017) results, *F_ROH_* provided the most accurate estimates of ID, whereas *F^III^* (called *F_alt_* by Nietlisbach et al., 2019) produced substantial upwardly biased estimates. Thus, these sets of simulations showed contradictory results.

The scenarios considered by Yengo et al. (2017) and Nietlisbach et al. (2019), however, were also very different in terms of population sizes and intensities of selection. For example, Nietlisbach et al. (2019) considered a subdivided population consisting of 30 demes of 200 individuals each connected by migration, assuming deleterious mutations with homozygous fitness effects exponentially distributed with mean −0.03. In contrast, Yengo et al. (2017) used in their simulations real human genotypic data from 10,000 unrelated individuals, and dominance effects were simulated assuming they were inversely proportional to the allele frequency variance and, therefore, with a potentially very large value.

Here, we carried out another set of genetic explicit simulations considering simplistic scenarios to evaluate the relative performance of different genomic marker *F* measures on the estimation of the rate of inbreeding depression. Our results show that, for low effective population sizes (*N* = 100), where individuals are expected to be more related to each other and where natural selection is, in general, relatively less intense, *F_ROH_* provides the most accurate estimates of ID, whereas *F^III^* provides overestimations, in full agreement with the results of Nietlisbach et al. (2019). However, for large effective population sizes (*N* ≥ 1,000), where individuals tend to be less related and deleterious alleles are expected to be at lower frequencies, *F^III^* provides almost unbiased average estimations of ID, in agreement with the results of Yengo et al. (2017), although with a great noise, whereas *F_ROH_* can produce slightly downwardly biased average estimates of ID, also with a great noise.

## 2 METHODS

### 2.1 Simulation procedure and parameters

Time‐forward individually based simulations were carried out of a diploid monoecious population of constant size *N* (100, 500, 1,000, 5,000 and 10,000) individuals with random mating. A modified version of the program SLIM (Messer, 2013) was used in which mutations with effect on fitness have a pleiotropic effect on a quantitative trait (Caballero, Tenesa, & Keightley, 2015). A single genome sequence of 100 Mb, initially devoid of variation, was assumed where mutations appear at a rate *U* per haploid genome and generation chosen to produce about 30,000 SNPs in the final generation for all population sizes. The rate of recombination between consecutive positions was assumed to be *c* = 10^−8^, which implies an average rate of recombination of 1 cM per Megabase. Thus, the genome length sequence (100 Mb) and the genetic length (1 M) are typical of a mammalian chromosome. Simulations were run for 10*N* discrete generations (5*N* in the case of *N* = 10,000). Ninety‐five per cent of mutations were assumed to be neutral, the remainder having an effect on a quantitative trait (QTL) and a pleiotropic effect on fitness. The genotypic values for the wild‐type homozygote, the heterozygote and the mutant homozygote were 0, *ah* and *a* for the quantitative trait, and 1, 1 + *sh* and 1 + *s* for fitness, where a constant dominance coefficient of *h* = 0.2 was assumed. Multilocus genotypic values assumed a multiplicative model for fitness and an additive model for the quantitative trait across loci. To minimize the noise, phenotypic values for the quantitative trait were assumed to equal genotypic values; that is, no environmental error was added to the phenotypic values. Values of *a* and *s* were obtained from a bivariate gamma distribution with mean effect −0.03 and shape parameter *β* = 1 (i.e., following an exponential distribution). A correlation (*ρ*) between *a* and *s* values was generally assumed to be one, that is, the trait is fitness itself, but *a* values were scaled so that the amount of ID was about the same for all population sizes considered (about 1% decrease in the mean per 1% increase in inbreeding or an inbreeding load of about one haploid lethal equivalent). Because mutations always reduced the value of the trait and were partially recessive, the model implied directional dominance, that is, homozygous recessive genotypes always reduced the trait value, thus generating ID for the trait. Except for the impact of purifying selection on deleterious mutations, the simulated populations were Wright–Fisher ideal populations so that the population size (*N*) approximately equals the effective population size.

For the population sizes of *N* = 100, 1,000 and 10,000, additional simulations were run considering all previous parameters (default scenario) with the following changes: (a) a one‐order higher (*c* = 10^−7^) or lower (*c* = 10^−9^) recombination rate between consecutive genomic positions. (b) A density of SNPs one quarter of that assumed previously, obtained by reducing to a quarter the mutation rate per generation, that is *U*/4. (c) A percentage of 10% (instead of 5%) of mutations affecting the trait and fitness. (d) An average effect of mutations on fitness of *s* = −0.1 instead of −0.03. (e) A distribution of effects for fitness and the trait with shape parameter for the gamma distribution of *β* = 0.1 or 2 instead of one. (f) A coefficient of dominance of mutations of *h* = 0 (complete recessive) instead of 0.2. (g) A correlation between fitness (*s*) effects and effects for the quantitative trait (*a*) of *ρ* = 0.5 instead of one. For these scenarios, the rate of inbreeding depression widely varied between 0.2% and 12% decrease in the mean per 1% increase in inbreeding, depending on the scenario and population size.

For all scenarios, the genotypes of all individuals of the population in the last simulated generation were obtained and the expected ID was quantified by the sum of 2*dpq* values for all segregating QTLs (Morton, Crow, & Muller, 1956), where *p* and *q* are the frequencies of the wild and mutant allele, respectively, and *d* = *a* (*h*–½) is the dominance effect. An estimate of the rate of inbreeding depression was also obtained by making all individuals homozygous (inbreeding coefficient *F* = 1) and calculating the change in the mean of the trait relative to that in the original individuals. The obtained values of ID coincided almost perfectly with their expectations.

The homozygous mutation load (Keller et al., 2011) was obtained as the number of homozygous mutations carried by individuals. Three different measures were obtained: (a) HML, the total number of homozygous mutations for neutral SNPs, that is, excluding QTLs; (b) HML_MAF_, the number of homozygous mutations for neutral SNPs with minor allele frequency lower than or equal to 0.05 (this is the definition applied by Keller et al., 2011); (c) HML_QTL_, the number of homozygous mutations for QTLs.

### 2.2 Measures of inbreeding coefficients and estimates of the rate of inbreeding depression

From the last generation of each simulation, the files *map* and *ped* were generated with all individuals of the population (to avoid the confounding effect of sampling) and all neutral segregating SNPs (i.e., QTLs were removed). No MAF pruning was made to the data. The following measures of the coefficient of inbreeding of each individual were then obtained with the programs PLINK (Purcell et al., 2007) and GCTA (Yang et al., 2011):

Estimators *F^I^*, *F^II^*, and *F^III^* provided by GCTAv1.93.0 with the –*ibc* option:

(1) $$F^{I}=\frac{1}{S}\sum_{k=1}^{S}\left(\frac{{\left(x_{k}‐2p_{k}\right)}^{2}}{2p_{k}\left(1‐p_{k}\right)}‐1\right),$$

(2) $$F^{\mathrm{II}}=1‐\frac{1}{S}\sum_{k=1}^{S}\frac{x_{k}(2‐x_{k})}{2p_{k}(1‐p_{k})},$$

(3) $$F^{\mathrm{III}}=\frac{1}{S}\sum_{k=1}^{S}\frac{x_{k}^{2}‐\left(1+2p_{k}\right)x_{k}+2p_{k}^{2}}{2p_{k}\left(1‐p_{k}\right)},$$

where *S* is the total number of markers, *x_k_* is the number of minor alleles of marker *k* (i.e., 0, 1 or 2 copies), and *p_k_* is the current frequency of the minor allele in the population.

The estimator *F^I^* is that proposed by VanRaden (2008) and referred to as VanRaden2. The estimator *F^III^* was called *F_alt_* by Keller et al. (2011) and Nietlisbach et al. (2019), *F_GRM_* by Huisman, Kruuk, Ellis, Clutton‐Brock, and Pemberton (2016) and *F_UNI_* by Yengo et al. (2017) and Alemu et al. (2020). For this measure of *F*, homozygous genotypes are weighted by the inverse of their allele frequencies (Yang et al., 2011), thus giving more weight to rare alleles. *F^III^* can also be obtained as $1‐\sum_{k=1}^{S}\delta /S$ (Keller et al., 2011) (with opposite sign), where *δ* equals 1/*p_k_* for homozygotes of the minor allele, 1/(1 – *p_k_*) for homozygotes of the major allele and zero for the heterozygotes, thus explicitly showing the larger weight given to rare rather than to common alleles.

The estimator *F_HOM_* is a measure of the deviation from Hardy–Weinberg proportions and is obtained by PLINK1.9 with the *–het* option.

(4) $$F_{\mathrm{HOM}}=1‐\frac{\sum_{k=1}^{S}x_{k}\left(2‐x_{k}\right)}{\sum_{k=1}^{S}2p_{k}\left(1‐p_{k}\right)}$$

Both *F_HOM_* and *F^II^* are based on the scaled difference between the observed (O[H]) and expected (E[H]) frequency of homozygotes, that is, (O[H] – E[H])/ (1 – E[H]), although the summation over markers is made differently in each case.

Finally, the estimator based on runs of homozygosity (*F_ROH_*) was obtained using PLINK1.9 with the default options: a minimum of 100 SNPs, at least 1 SNP per 50 Kb, and a scanning window of 50 SNPs. We considered runs of lengths larger than 0.1 Mb (*F_ROH_*_‐0.1_), 1 Mb (*F_ROH_*_‐1_) or 5 Mb (*F_ROH_*_‐5_), after removing highly linked SNPs (*r* ^2^ > .9) with the *‐‐indep‐pairwise 50 5 0.9* PLINK option, as recommended by Howrigan, Simonson, and Keller (2011).

An explanation of the rationale and relationship between the estimators *F^I^*, *F^II^*, *F^III^* and *F_HOM_* is given in the Supplementary Appendix. The inbreeding coefficient of an individual (*F*), that is, the probability of identity by descent of the two alleles at a locus, is a concept relative to a (sometimes arbitrary) reference base population (e.g., an earlier generation of the population). If the allele frequencies of the reference generation are considered in the estimators, these are expected to measure the inbreeding coefficient (IBD) relative to that reference generation, at least when all loci are at linkage equilibrium (see Supplementary Appendix). Unfortunately, the reference generation frequencies are usually unknown and the estimates are obtained assuming the current generation allele frequencies. In this case, the measures of *F* from (1), (2), (3), (4) refer to the deviations of the frequencies of homozygotes from those expected under Hardy–Weinberg expectations or the correlation between the alleles carried by individuals. Thus, they take positive or negative values, generally implying an excess or a defect, respectively, of homozygotes. In contrast, the measures obtained by *F_ROH_* take only positive values (from zero to one) as they are expected to include genomic segments of homozygosity produced by IBD.

The estimated ID was obtained as the slope of the linear regression of the phenotypic values (*Phe*) of individuals on their different *F* measures. All individuals of the population were used in this regression to avoid biases due to sampling errors. In addition, as was mentioned above, phenotypic values were taken with no error, so that any deviation of the estimated ID from its true value should be ascribed to the different properties of the molecular measures of *F*.

Simulations were repeated between 100 and 1,000 times depending on the population size considered. For each replicate, the estimated ID obtained with each *F* measure was compared with the expected (true) ID, calculating the distribution of deviations from the expected value and the root‐mean‐square error (RMSE), a combined measure of accuracy and precision. Pearson correlations between all *F* values and between these and the phenotypic values of individuals (*Phe*) and the different homozygous mutations loads (*HML*, *HML_MAF_* and *HML_QTL_*) were also obtained and averaged across replicates.

### 2.3 Alternative set of simulations

To double‐check the main results obtained, additional time‐forward individually based simulations were performed with an in‐house C program alternative to SLIM, modified from Caballero, Bravo, and Wang (2017). In this case, populations of size *N* = 100, 1,000 and 5,000 were run for 1,000 (*N* = 100) or 10,000 (*N* ≥ 1,000) discrete generations, assuming a 60 Kb genome sequence of 1 Morgan genetic length and a mutation rate adjusted to produce up to 30,000 SNPs. Thus, the number of SNPs and the genetic length were similar to those of the previous simulations. A 10% proportion of mutations were assumed to be deleterious with average selection and dominance coefficients of *s* = −0.03 and *h* = 0.2, respectively. Selection coefficients for mutations were obtained, as before, from an exponential distribution, but dominance coefficients were assumed to be variable, with an inversely proportional relationship with selection coefficients following the model proposed by Caballero and Keightley (1994) (see also Caballero, 2020, p. 159). Values of the true ID and those estimated with the different *F* values were obtained from each of the last 200 generations of each replicated simulation, considering the whole population. Given the short genome sequence assumed in these simulations, ROH segments larger than 1 Kb were considered.

## 3 RESULTS

Table 1 shows the mean and variance of all *F* measures in the default scenario for three population sizes. SNP‐by‐SNP‐based *F* measures using Hardy–Weinberg deviations or correlations between alleles (*F^I^*, *F^II^*, *F^III^* and *F_HOM_*) have virtually the same mean, as expected (see Supplementary Appendix), but *F^III^* and *F_HOM_* show a lower variance, particularly *F^III^*. Mean and variances of *F_ROH_* estimates are lower for increasing fragment lengths. Table 2 presents the average correlations between *F* measures. *F^I^* has a strong negative correlation with *F^II^*, as suggested from their different subtracting terms (see Equations 1 and 2), and generally low correlations with the other *F* measures. *F^II^* also shows intermediate (for small *N*) or low correlations (for large *N*) with the other measures, but larger than those for *F^I^*. Measures *F^III^*, *F_HOM_* and *F_ROH_* show rather large correlations with one another, decreasing for larger population sizes.

**Table 1:** Mean and variance of different genomic measures of the inbreeding coefficient (*F*; see text for definitions), averaged over replicates, for different population sizes (*N*)

	*F^I^*	*F^II^*	*F^III^*	*F_HOM_*	*F_ROH−_*_0.1_	*F_ROH_*_−1_	*F_ROH−_*_5_
Mean							
*N*= 100	−0.0057	−0.0053	−0.0053	−0.0052	0.1398	0.1120	0.0430
*N* = 1,000	−0.0006	−0.0005	−0.0005	−0.0006	0.1720	0.0712	0.0108
*N* = 10,000	−0.00004	−0.00004	−0.00004	−0.00004	0.0784	0.0072	0.0010
Variance							
*N* = 100	0.0424	0.0435	0.0077	0.0250	0.0130	0.0130	0.0110
*N* = 1,000	0.0264	0.0266	0.0011	0.0039	0.0025	0.0026	0.0018
*N* = 10,000	0.0660	0.0660	0.0002	0.0007	0.0005	0.0003	0.0002

Note

Standard errors of means and variances are below 0.002 (*N* = 100) and 0.003 (*N* = 1,000 and 10,000).

[Correction added on 19 January 2021, after first online publication: table 1 has been modified.]

**Table 2:** Correlation between different genomic measures of the inbreeding coefficient (*F*; see text for definitions), averaged over replicates, for different population sizes (*N*)

	*F^II^*	*F^III^*	*F_HOM_*	*F_ROH_*_−0.1_	*F_ROH_*_−1_	*F_ROH_*_−5_
*N* = 100						
*F^I^*	−0.6438	0.3922	0.1544	0.2038	0.2083	0.2206
*F^II^*		0.4279	0.5661	0.5193	0.5091	0.4500
*F^III^*			0.8786	0.8775	0.8694	0.8046
*F_HOM_*				0.8526	0.8244	0.6946
*F_ROH_*_−0.1_					0.9868	0.8507
*F_ROH_*_−1_						0.8785
*N* = 1,000						
*F^I^*	−0.9171	0.1933	0.0690	0.0829	0.1000	0.1172
*F^II^*		0.2076	0.2754	0.2555	0.2450	0.2144
*F^III^*			0.8587	0.8427	0.8577	0.8190
*F_HOM_*				0.8810	0.8192	0.6576
*F_ROH_*_−0.1_					0.8893	0.6845
*F_ROH_*_−1_						0.7607
*N* = 10,000						
*F^I^*	−0.9946	0.0501	−0.0160	0.0114	0.0324	0.0316
*F^II^*		0.0525	0.0932	0.0619	0.0512	0.0450
*F^III^*			0.7506	0.7125	0.8102	0.7366
*F_HOM_*				0.7138	0.6075	0.4879
*F_ROH_*_−0.1_					0.6747	0.5240
*F_ROH_*_−1_						0.7681

Note

Standard errors are below 0.005 (*N* = 100), 0.003 (*N* = 1,000) and 0.006 (*N* = 10,000).

**Figure 1:**
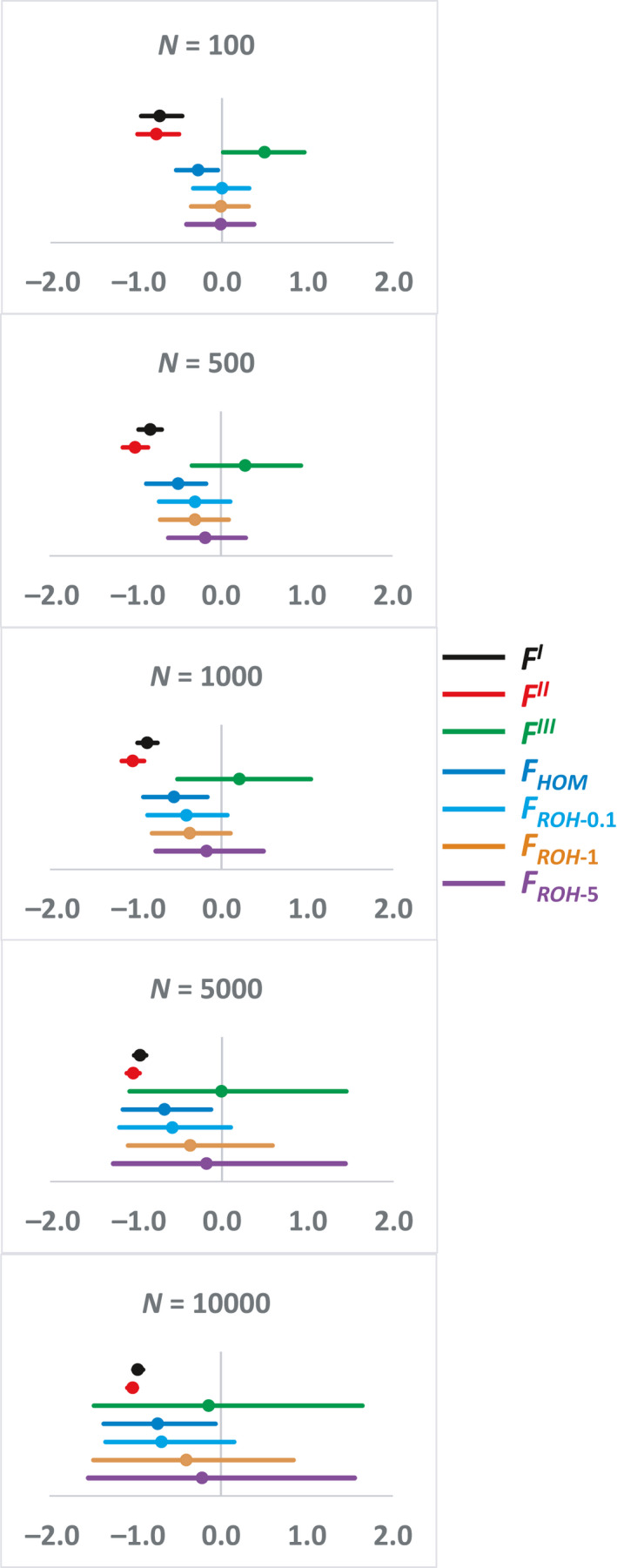
Proportional deviation of the estimates of the rate of inbreeding depression (ID) obtained with different measures of the inbreeding coefficient with marker data (see text), with respect to the true simulated ID. The dot is the mean deviation, and the bar indicates the 95% of the distribution of simulated replicates. Simulated parameters: population size *N*; genome sequence of 100 Mb run for 10*N* discrete generations (5*N* in the case of *N* = 10,000); rate of recombination between consecutive positions *c* = 10^−8^; mutation rate per haploid genome and generation *U* chosen to produce about 30,000 SNPs in the final generation for all population sizes; 95% of mutations assumed to be neutral, the remainder having an effect on a quantitative trait (QTL) and a pleiotropic effect on fitness; homozygous effects for the trait (*a*) and fitness (*s*) obtained from a bivariate gamma distribution with mean effect −0.03 and shape parameter *β* = 1; the mean homozygous effect for the trait (*a*) was adjusted to obtain a true inbreeding depression rate of about 1 (one per cent decline in mean per one per cent increase in inbreeding or an inbreeding load of about one haploid lethal equivalent) for all population sizes; dominance coefficient *h* = 0.2; correlation *ρ* = 1 between *a* and *s* values

Figure 1 shows the proportional deviations of the estimated ID values obtained with different neutral molecular *F* measures with respect to the true values, that is, (EstID – TrueID)/ TrueID, where EstID and TrueID are the estimated and true ID, respectively. Thus, a value of, say + 1, implies an overestimation of ID by 100% and a value of −1 an underestimation of ID by 100%. The bars exclude 2.5% of the extreme deviations at each side and the dot is the arithmetic mean of the deviations. The measures *F^I^* and *F^II^* provide always very biased estimates (underestimations) of ID, and this is also the case, although to a less extent, for *F_HOM_*. For small population size (*N* = 100), *F^III^* produces upwardly biased estimates whereas estimates with *F_ROH_* are unbiased, irrespective of the ROH minimum length assumed. However, as the population size *N* is increased, *F_ROH_* estimates of ID can become underestimations, particularly if short fragment lengths are included in the analysis, whereas those from *F^III^* become more and more accurate on average, although with a large variation across replicates. For the largest population size (*N* = 10,000), both *F_ROH_* and *F^III^* incur in a slight underestimation of ID on average, but with a great variation. Thus, *F^III^* transits from providing overestimations of ID for small population sizes to slight underestimations for large ones. A representation of the particular deviations observed for each simulated replicate with different population sizes is shown in Figure S1.

**Figure 2:**
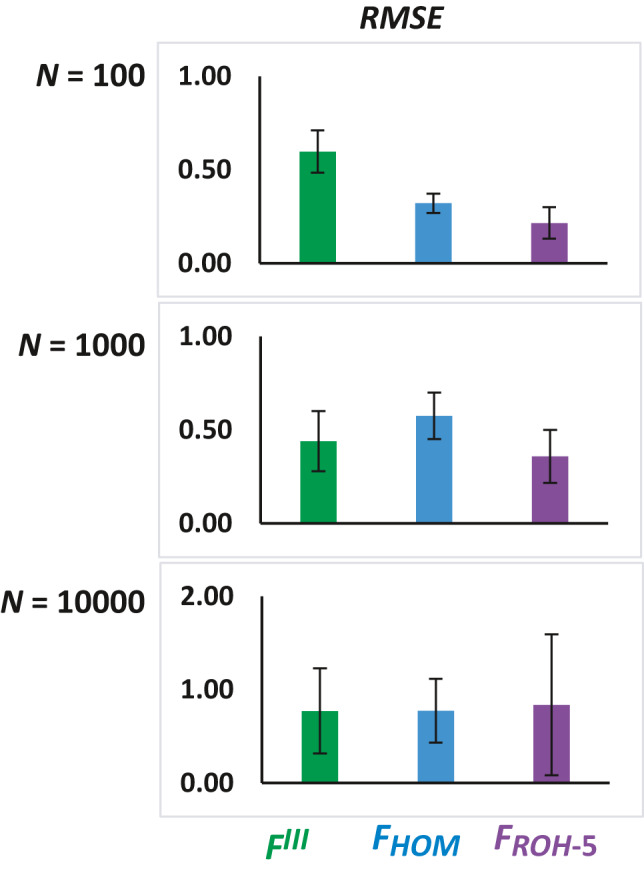
Root‐mean‐square error (RMSE) of estimates of the rate of inbreeding depression obtained from three measures of the individual inbreeding coefficient (*F^III^*, *F_HOM_* and F_ROH‐5_; see text). Results refer to the default parameters as in Figure 1. Bars indicate one standard error of the mean

For *N* = 100, the average root‐mean‐square error (RMSE) of the estimates of ID obtained with *F_ROH_*_‐5_ (0.216 ± 0.047) is lower than that obtained with *F^III^* (0.597 ± 0.097) (Figure 2). However, for larger population sizes, this difference disappears, for example for *N* = 10,000, RMSE *F_ROH_*_‐5_ = 0.837 ± 0.255 and RMSE *F^III^* = 0.772 ± 0.231.

**Figure 3:**
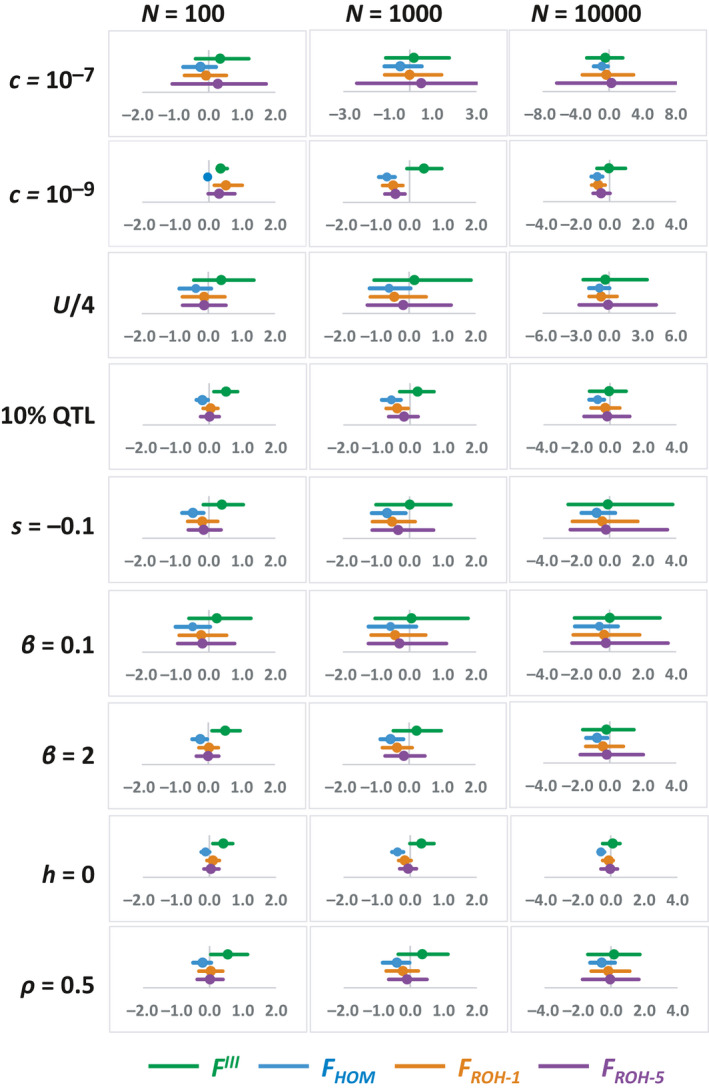
Proportional deviation of the estimates of the rate of inbreeding depression (ID) obtained with different measures of the inbreeding coefficient with marker data (see text), with respect to the true simulated ID. Simulations assume different population sizes (*N*). The dot is the mean deviation and the bar indicates the 95% of the distribution of simulated replicates. The simulation parameters are the same as for Figure 1 (default parameters), with the following changes: (Row 1) recombination rate between positions *c* = 10^−7^. (Row 2) recombination rate between positions *c* = 10^−9^. (Row 3) density of SNPs one quarter of that assumed in Figure 1. (Row 4) 10% of mutations affecting the trait and fitness. (Row 5) Average effect of mutations on fitness of −0.1. (Row 6) Distribution of effects for fitness and the trait with shape parameter for the gamma distribution of *β* = 0.1. (Row 7) Distribution of effects for fitness and the trait with shape parameter for the gamma distribution of *β* = 2. (Row 8) coefficient of dominance of mutations of *h* = 0. (Row 9) correlation between fitness (*s*) effects and effects for the quantitative trait (*a*) of *ρ* = 0.5

Figure 3 shows results analogous to those of Figure 1 for different parameters alternative to those of the default scenario. These include different recombination rates, density of SNPs, intensity of selection, distribution of allelic effects and degree of recessiveness. Rates of inbreeding depression obtained using *F^I^* and *F^II^* give underestimations for all scenarios and are not shown. Thus, results are only given in the figure for *F^III^*, *F_HOM_*, *F_ROH_*_‐1_ and *F_ROH_*_‐5_. Although some quantitative differences can be observed for the different scenarios, the main conclusions reached above generally hold. *F^III^* gives overestimations of ID in all scenarios for *N* = 100, becoming almost unbiased, on average, for *N* = 10,000. *F_HOM_* usually underestimates ID. *F_ROH_*_‐5_ provides generally good estimates of ID with a tendency to a slight underestimation. Averaging results from all scenarios gives an outcome very similar to that of the default scenario (cf. Figure 1 and Figure S2). In addition, the corresponding results using a simulation program alternative to SLIM (Figure S3) gives the same general picture. Estimates of ID from *F_ROH_* (with ROH > 1 Kb) are nearly unbiased for *N* = 100 but imply underestimations for larger *N*, whereas the overestimation incurred by *F^III^* is reduced as *N* is increased. In this case, the error bars are very short because each simulated value obtained is the average of 200 estimations carried out in the last 200 generations of each simulation.

Table 3 gives the correlation between the phenotypic values of individuals (*Phe*) and the different measures of the homozygous mutation load (HML). All correlations are diminished as the population size is increased, as expected. In all cases, also as expected, *Phe* has the largest correlation with *HML_QTL_*. For small population sizes (*N* = 100), *Phe* has a larger correlation with *HML* than with *HML_MAF_*. In contrast, for larger *N*, *Phe* is more correlated with *HML_MAF_* than with *HML*, suggesting that the phenotype of individuals can be ascribed more strongly to rare homozygous alleles. In fact, as shown in Figure S4, the larger the population size, the lower the QTL frequencies and the larger the contribution to ID by rare alleles, as would be expected. Nevertheless, for large *N* (particularly for *N* = 10,000), all correlations between the different values of HML and *Phe* are rather low, indicating that the homozygous mutation load is a poor proxy of fitness in large populations.

**Table 3:** Correlation between phenotypic values (*Phe*) for the quantitative trait and the homozygous mutation load, obtained as the number of mutant homozygotes carried by individuals considering all neutral SNPs (*HML*), neutral SNPs with MAF < 0.05 (*HML_MAF_*), and quantitative trait loci (*HML_QTL_*), for different population sizes (*N*)

	*HML*	*HML_MAF_*	*HML_QTL_*
*N* = 100			
*Phe*	0.6652	0.4538	0.7930
*HML*		0.4711	0.8949
*HML_MAF_*			0.4771
*N* = 1,000			
*Phe*	0.1237	0.1723	0.3020
*HML*		0.6226	0.3694
*HML_MAF_*			0.3270
*N* = 10,000			
*Phe*	0.0107	0.0158	0.0712
*HML*		0.5018	0.0600
*HML_MAF_*			0.0663

Note

Standard errors are below 0.005 (*N* = 100) and 0.002 (*N* = 1,000 and 10,000).

**Figure 4:**
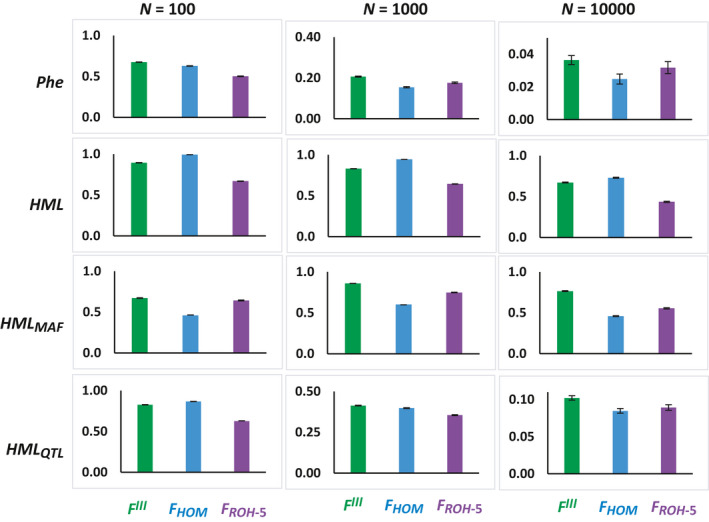
Correlation between the individual values of the genomic measures of the inbreeding coefficient (*F^III^*, *F_HOM_* and F_ROH‐5_; see text) and the individual phenotypic value for the quantitative trait (*Phe*), the total homozygous load for neutral SNPs (*HML*), the homozygous load for neutral SNPs with MAF ≤ 0.05 (*HML_MAF_*) and the homozygous load for QTLs (*HML_QTL_*). Bars indicate one standard error of the mean

Finally, Figure 4 shows the correlation between the *F* measures and the phenotypic value (*Phe*) of individuals and the homozygous mutations loads (*HML*). *F^III^* has the largest correlation with *Phe* and *HML_MAF_* for all values of *N* and with *HML_QTL_* for *N* ≥ 1,000. The largest correlation with HML is achieved by *F_HOM_*, as expected.

## 4 DISCUSSION

Inbreeding depression is a key issue for explaining the evolution of populations and to carry out the management and conservation of wild and domestic species (Lynch & Walsh, 1998). The increasing availability of dense molecular markers (SNPs) for many species has allowed the prediction of the relationships among individuals in the absence of pedigree records. In fact, genomic measures of *F* can be more useful than pedigree estimates, as they provide realized rather than expected genomic relationships (Kardos et al., 2016; Keller et al., 2011). These estimates of genomic inbreeding can, in turn, be used to estimate the rate of inbreeding depression. If the allele frequencies of many loci at Hardy–Weinberg and linkage equilibrium were known without error at a given previous generation of the population and these frequencies were included in the different SNP‐by‐SNP *F* measures in the current generation, their expected values would provide the true mean inbreeding coefficient (IBD) referred to that previous generation (i.e., the *F* value that would be obtained from the pedigree) (see Supplementary Appendix). The usual scenario, however, is that only the current allele frequencies are available to be used in the different SNP‐by‐SNP *F* measures and these provide deviations of the genotypic frequencies from their HW expectations. Estimates from genomic segments of homozygosity (*F_ROH_*) are intended to look for events of IBD. However, not all ROH involve IBD and the measures obtained strongly depend on the length of the fragments considered. Thus, it is unclear which *F* measures from molecular data are the most appropriate to estimate the rate of inbreeding depression. While the simulation results of Yengo et al. (2017) supported the use of SNP‐by‐SNP‐based *F* estimates with more weight for rare alleles, those of Nietlisbach et al. (2019) pointed towards the use of ROH‐based *F* estimates. Our results seem to reconcile these contrasting results and suggest that the recommendation to be made strongly depends on the particular population considered.

Nietlisbach et al. (2019) compared different *F* measures carrying out genetically explicit simulations of a metapopulation under purifying selection against deleterious mutations. The metapopulation included 30 demes of 200 individuals each connected by migration (1.2 migrants per deme and generation) and was run for 5,000 discrete generations. Viability selection was considered on a genome mimicking that of the great tit, with around 50,000 neutral loci and 2,500 deleterious loci acting multiplicatively. Homozygous effects were exponentially distributed with mean *s* = −0.03 and a mean dominance coefficient of *h* = 0.18 (following estimated parameters from Wang, Hill, Charlesworth, & Charlesworth, 1999). To perform their simulations, they used a binary viability model and compared the different methods that can be used to estimate ID in this type of models. Deleterious variants were excluded from the analysis of genomic inbreeding and apparently no filtering for MAF was applied to the data. ROH with length > 1 Mb were obtained using sliding windows of 50 SNPs with steps of 5, allowing up to one heterozygote per segment and removing SNPs with high linkage disequilibrium (*r* ^2^ > .9). The main conclusion obtained by Nietlisbach et al. (2019) was that, under their simulated scenario, *F_ROH_* provides unbiased average estimations of ID, whereas *F^III^* gives severe average overestimations (about +0.7 in the scale given in our Figure 1), and *F_HOM_* a slight underestimation (about −0.2). Their results also showed that the variation between the estimates of ID was smaller by using *F_ROH_* than by using *F^III^* and those from *F_HOM_* had the lowest variation (see Nietlisbach et al., 2019, Figure 2). In addition, the RMSE of ID estimates obtained from *F_HOM_*, *F_ROH_* and *F^III^* were 0.86, 1.01 and 2.02, respectively. Thus, *F^III^* estimates of ID showed about twice as large RMSE as *F_ROH_* ones.

Yengo et al. (2017), in contrast, used a different simulation approach, by considering human genotypic data of 10,000 unrelated individuals (pairwise genetic relationship < 0.05) and assigning phenotypic values with a model for which dominance effects were assumed to be constant or a function of the inverse of the variance of the allele frequency. In these simulations, therefore, a much larger population size was assumed than for the individual demes of Nietlisbach et al. (2019), and dominance effects could be very substantial for loci with rare alleles. The analysis of inbreeding measures was carried out assuming a MAF of 0.05 and ROH fragments were obtained either pruning high linkage disequilibrium (LD) SNPs or considering only fragments > 1.5 Mb. Yengo et al. (2017) considered different scenarios, such that causal variants could be enriched in random, high‐ or low‐LD genome regions. In the random LD scenario (the causal variants are a random subset of all SNPs) with dominance effects inversely related to the variance of the allele frequencies, *F^III^* was found to provide unbiased estimates of ID, *F_HOM_* a slight underestimation and *F_ROH_* (with ROH lengths > 1.5Mb) a large average overestimation of about 90% of ID (see Yengo et al., 2017, Figure 1b), that is, a value of about +0.9 in the scale of our Figure 1. They also found that the estimates of ID from *F_ROH_* had about a threefold larger standard error than those from *F^III^*. Additionally, they found that both *F^III^* and *F_HOM_* produced overestimates of ID when QTLs are enriched in high‐LD regions, and underestimates of ID when enriched in low‐LD regions, and that these biases could be corrected or reduced if LD score and MAF stratification were applied. Population stratification also produced biases in estimates of ID from *F_HOM_* and *F_ROH_*, which were not observed in those from *F^III^*. In accordance with their simulated results, the estimations of ID over 25 human quantitative traits were consistently larger when obtained from *F_ROH_* measures than from *F^III^* ones. However, more significant cases of ID were detected with *F^III^* than with *F_ROH_*.

Our simulations consider some parameters similar to those assumed by Nietlisbach et al. (2019), such as the average selection coefficient and dominance, but assume a single undivided population of varying size. We also considered a simple additive multilocus model for a quantitative trait rather than a binary viability model, so that a log scale is not necessary to be applied and the regression of raw phenotypic values on the predicted *F* provides a direct estimate of the inbreeding depression rate. Our simulations for *N* = 100 show no bias of the average ID when *F_ROH_* is used, irrespective of whether short (>0.1 or >1 Mb) or long (>5 Mb) ROH are considered (Figure 1). *F_HOM_* underestimates ID by a fraction of about −0.3, whereas *F^III^* overestimates it by a fraction of about +0.5. These results are actually very similar to those obtained by Nietlisbach et al. (2019), that is −0.2 and +0.7, respectively. In addition, for *N* = 100 we found that the RMSE of ID estimates from *F^III^* measures were three times larger than those from *F_ROH_* ones (Figure 2), in concordance with a twofold difference in the same direction observed by Nietlisbach et al. (2019). We observed, however, that estimates of ID from *F_HOM_* for *N* = 100 had a RMSE larger than those from *F_ROH_* (Figure 2), whereas Nietlisbach et al. (2019) results showed a RMSE for *F_HOM_* slightly lower than that from *F_ROH_*. We should note, however, that apparently, Nietlisbach et al. (2019) did not consider estimates of ID below zero (see their Figure 2), what may explain this difference between both studies.

In contrast, our results are rather different from those of Nietlisbach et al. (2019) when larger population sizes are assumed. In this scenario, estimates of ID by *F_ROH_* can become downwardly biased in our simulations, particularly if short ROH are considered (Figure 1). For example, with ROH of length > 1 Mb the underestimation of ID is in a fraction of about −0.01, −0.30, −0.37, −0.37 and −0.40 for increasing population sizes, from 100 to 10,000. If large fragments are used in the calculations (>5 Mb), the bias is lower (−0.01, −0.18, −0.17, −0.18 and −0.22 for increasing *N*). The increased underestimation of ID when short ROH are considered may occur because some short ROH may not reflect IBD, as suggested by Pryce et al. (2014), and thus, *F_ROH_* overestimates the true genomic inbreeding (cf. mean values of *F_ROH_*_‐0.1_, *F_ROH_*_‐1_ and *F_ROH_*_‐5_ in Table 1). However, it can also be argued that long ROH only account for recent inbreeding whereas measures of *F* including also short ROH would incorporate more ancient inbreeding, what may contribute to values of short‐ROH‐*F* measures larger than those from long ones.

The increase in population size also results in a reduction of the average bias of the estimated ID using *F^III^*, whose bias becomes 0.5, 0.28, 0.21, −0.002 and −0.14 for increasing values of *N*. Thus, average estimates of ID with *F^III^* for large population sizes are basically unbiased or slightly so, in agreement with the main result obtained by Yengo et al. (2017). Our results do not agree with those of Yengo et al. (2017), however, in relation with the estimation of ID using *F_ROH_*, as they obtained overestimations by a fraction +0.9 whereas we obtained almost unbiased estimates or slight underestimations for large *N*. In fact, Yengo et al. (2017) found generally much larger estimates of ID for 25 human quantitative traits when *F_ROH_* was used than when *F^III^* was used. Kardos et al. (2018) argued that the *F_ROH_* measures considered by Yengo et al. (2017) only accounted for long ROH and, thus, for only recent inbreeding. If short ROH and thus more ancient inbreeding would have been incorporated, this could have resulted in larger estimates of *F* and lower ID values. Nevertheless, the larger the population size, the larger the noise in the estimates of ID both from *F^III^* and *F_ROH_*, pointing towards the difficulties of estimating the rate of inbreeding depression in large outbred populations. We observed that variation in the estimates of ID from *F^III^* and *F_ROH_* were similar in the case of *N* = 10,000 (see Figure 1) and that the RMSE of these estimates were also about the same for these two *F* measures (see Figure 2 for *N* = 10,000). This contrasts with the result of Yengo et al. (2017), which indicated that the estimates of ID from *F_ROH_* had a threefold larger standard error than those from *F^III^*. It must be noted, however, that our simulations refer to a simplistic model of a constant population size, with uniform LD along the genome and without any source of stratification, which can differ from the complex structure of the human genomes and populations analysed by Yengo et al. (2017). Thus, there may be a source of unknown factors that could be contributing to the differences between the results of both studies.

We obtained estimates of the mean, variance and correlation between the different genomic *F* measures and between these and the homozygous mutation load (HML), suggested to be a proxy of ID (Keller et al., 2011). The mean of the genomic measures *F^I^*, *F^II^*, *F^III^* and *F_HOM_* is basically the same (Table 1) and close to the value of the deviation from Hardy–Weinberg proportions expected in a panmictic population, −1/2*N* (Kimura & Crow, 1963; Robertson, 1965). The correlation results indicate that *F^I^* (VanRaden2) and *F^II^* measures of inbreeding are poorly correlated with the other measures (Table 2) and are poor estimators of ID (Figure 1 and Figure S3). They should therefore be disregarded in the estimation of ID. In addition, these estimators show a larger variance than the other SNP‐by‐SNP‐based measures of inbreeding, *F_HOM_* and *F^III^*. The latter shows the lowest variance, as demonstrated by Yengo et al. (2018). Correlations between *F^III^*, *F_HOM_* and *F_ROH_*, however, are generally high, as observed in empirical studies (e.g., Bérénos, Ellis, Pilkington, & Pemberton, 2016). Estimates of ID from *F_HOM_*, however, are also generally biased (Figure 1 and Figures S3), so this *F* measure should not be used either to estimate ID.

We found that *F^III^* is the measure of genomic inbreeding showing the largest correlation with the phenotypic values of individuals (Figure 4), as well as with *HML_MAF_* (the definition of homozygous mutation load from Keller et al., 2011), and the HML applied to QTLs (*HML_QTL_*) for large populations. Thus, our results suggest that *F^III^* is the *F* statistic more related with the load of deleterious recessive mutations, as also suggested by the analysis of genomic cattle data by Alemu et al. (2020). Some of these results contrast with those of Keller et al. (2011), who found that *F_ROH_* had the largest correlation with *HML_MAF_*, followed by *F^III^* and then *F_HOM_* (see Figure 7 of Keller et al., 2011), whereas we observed that the largest correlation is for *F^III^*, followed by *F_ROH_* and then *F_HOM_* (see Figure 4). However, the simulations of Keller et al. (2011) did not consider selection of any type. Purifying selection, as applied in our simulations, would imply some reduction in genetic variability across the genome, so that rare allele frequencies would get more relevance in the HML.

Regarding *F_ROH_*, we found that the lowest bias in the estimation of ID occurred when large ROH were considered (Figure 1 and Figure S3). This could be explained, as mentioned above, because short ROH may not fully reflect IBD, overestimating the true genomic inbreeding, but also because short ROH are less enriched in QTLs. Szpiech et al. (2013) showed that ROH in the human genome are enriched in deleterious mutations and that long ROH are more enriched than short ones. In cattle, however, the result is the opposite, with short ROH more enriched in deleterious mutations than long ones (Zhang et al., 2015). Our simulations show that, for the smallest population size (*N* = 100), the correlation between the phenotypic values (*Phe*) and *F_ROH_* is 0.66 ± 0.003 for *F_ROH_*_‐0.1_ and 0.58 ± 0.004 for *F_ROH_*_‐5_, in line with the cattle results. In contrast, for the largest population size (*N* = 10,000) the corresponding correlations are 0.012 ± 0.001 for *F_ROH_*_‐0.1_ and 0.017 ± 0.001 for *F_ROH_*_‐5_, in agreement with the human genome results. It is expected that cattle populations, heavily subjected to selection, have lower effective population sizes than human populations, so our results are consistent with the observations.

The *F* measure showing the largest correlation with the overall homozygosity mutation load (*HML*, considering all allele frequencies) was *F_HOM_*. Multilocus heterozygosity has been traditionally related to overall fitness, although with a poor correlation (Grueber, Waters, & Jamieson, 2011; Slate & Pemberton, 2002). This, however, has been mainly focused on few markers (e.g., microsatellites) and the possibility to explore the multilocus heterozygosity—fitness correlation with highly dense markers using *F_HOM_* or other measures of *F* opens new research areas.

In summary, our simulation results indicate that estimates of the rate of inbreeding depression from *F_ROH_* measures of inbreeding are appropriate for populations with low effective sizes but may lead to some underestimation for large ones, unless the ROH fragments considered are sufficiently long. Estimates of the rate of inbreeding depression from rare‐SNP‐by‐SNP‐based *F* measures (*F^III^*) are severely biased in populations of small effective size, but nearly unbiased in large ones, showing the largest relationship with the phenotypic values of individuals and the homozygous mutation load. This different performance of the *F* measures depending on the population size can respond to the expectation that small populations are subjected, in general, to less intense selection than large ones, although purging selection of the inbreeding load can be more effective in the former (García‐Dorado, 2012; López‐Cortegano, Vilas, Caballero, & García‐Dorado, 2016). The use of *F^I^* and *F^II^* measures of genomic inbreeding and, to a lesser extent *F_HOM_*, cannot be advised to estimate ID. For large effective population sizes, however, the estimation of the rate of inbreeding depression becomes rather difficult as all estimations are subjected to a high error.

## CONFLICT OF INTEREST

None declared.

## Supporting information

Supplementary Material

## Data Availability

All software and scripts used in the simulations are available in GitHub: https://github.com/armando‐caballero/Caballero‐et‐al.‐Evol‐Appli‐2020.
