# Supplementary material for: On the estimation of inbreeding depression using different measures of inbreeding from molecular markers
Source: Evol Appl. 2020 Oct 23;14(2):416–28. doi: 10.1111/eva.13126 (PMC7896712; doi:10.1111/eva.13126)
Supplement: Supplementary file 1 — Supplementary Material [file EVA-14-416-s001.docx]

**SUPPLEMENTARY MATERIAL**

**On the estimation of inbreeding depression using different measures of inbreeding from molecular markers**

**Armando Caballero, Beatriz Villanueva and Tom Druet**

**APPENDIX**

**Relationship between the different *F* estimators from molecular data**

The coefficient of inbreeding (*F*) of an individual was defined by Wright (1922) as the correlation between the alleles carried by individuals at a given locus. An alternative definition of *F* is the probability that the two uniting gametes to form an individual carry alleles at a given locus that are identical by descent (IBD; Malécot, 1948), i.e. both are copies of an allele carried by a common ancestor of the parents of the individual. While under the former definition *F* can take positive or negative values, for the latter it ranges between zero and one. The equivalence between both definitions was shown by Crow and Kimura (1970, p. 67). When the concept of IBD is extended to pairs of individuals, the coefficient of kinship or coancestry (*f*) (Malécot, 1948) is defined as the probability that two alleles at the same locus taken at random from two individuals are identical by descent, which can then be obtained for any pair of individuals *i* and *j* (*f_ij_*). The coancestry between two mating individuals is, by definition, the expected inbreeding of their offspring, and the self-coancestry of one individual is *f_ii_* = ½[*F_i_* + 1], so that the coefficient of inbreeding of an individual can be obtained from the self-coancestry as

*F_i_* = 2*f_ii_* –1. (A1)

When these concepts are applied to molecular markers, we can define the molecular inbreeding (or molecular homozygosity), and the molecular coancestry (or similarity coefficient). The coefficient of molecular inbreeding (*F_M,i_*) of an individual *i* for a given locus is 0 if the individual carries different alleles for the locus and 1 if these are identical. Analogously, the molecular coancestry (*f_M,ij_*) between two individuals *i* and *j* is calculated by comparing two by two the alleles of each individual. These coefficients are not proper inbreeding and coancestry coefficients in the sense that they do not account just for identity by descent, but identical alleles can arise from different ancestors (i.e. they are said to be identical in state, IIS). We will keep, however, the denomination of molecular inbreeding and molecular coancestry in analogy to the genealogical ones for the sake of clarity.

Consider, for simplicity, a single locus *k* with 2 alleles with population frequencies at a given reference (base) population *p_k_* and *q_k_* = 1 – *p_k_*, with genotypic frequencies at Hardy-Weinberg proportions. As deduced by Cockerham (1969), the expected molecular coancestry between two individuals for the locus is

$f_{M,ij}=f_{ij}+\left(1-f_{ij}\right)\left[p_{k}^{2}+q_{k}^{2}\right]$ (A2)

(see, e.g. Caballero, 2020, p. 73). The first term at the right-hand side of the equality is the probability that the two alleles taken from each individual are identical by descent (*f_ij_*), and the second term the probability that they are identical in state but not descent. Solving, we obtain an estimate of the genealogical coancestry from the molecular coancestry,

$\hat{f}_{ij}=\frac{f_{M,ij}-\left[p_{k}^{2}+q_{k}^{2}\right]}{2p_{k}q_{k}}$ . (A3)

Considering the self-coancestry of individual *i* (*f_ii_*) and applying Eqn. A1 (which also holds for the molecular estimates *F_M_* and *f_M_*), we obtain that the expected inbreeding coefficient of an individual can be estimated from the molecular data as

$\hat{F}_{i}=\frac{F_{M,i}-\left[p_{k}^{2}+q_{k}^{2}\right]}{2p_{k}q_{k}}$ (A4-a)

(Li and Horvitz, 1953). This is, therefore, an estimate of the deviation of the observed frequency of homozygotes (*F_M,i_*) from that expected under Hardy-Weinberg proportions with the base population frequencies.

Taking *x_k_* as the number of copies of an allele of locus *k* in an individual, i.e. *x_k_* = 0, 1 or 2 if the individual carries 0, 1 or 2 copies of the allele, and noting that *x_k_*(2 – *x_k_*) = 1 – *F_M,i_* (the observed frequency of homozygotes), we obtain

$\hat{F}_{i}=F^{II}=F_{HOM}=1-\frac{x_{k}\left(2-x_{k}\right)}{2p_{k}q_{k}}$ , (A4-b)

which equals the estimators *F^II^* (Eqn. 2 in the main text) and *F_HOM_* (Eqn. 4 in the main text) in the case of a single locus (*S* = 1). The difference between Eqns. 2 and 4 resides in how the summation is made over loci. The application of Eqn. A4 to the three genotypes at a locus is shown in the first row of Table A1, which is analogous to Table 1 of Zhang et al. (2015).

| Estimator | *x_k_* = 0 | *x_k_* = 1 | *x_k_* = 2 | Average |
| --- | --- | --- | --- | --- |
| A4 (*F^II^*, *F_HOM_*) | 1 | $1-\frac{1}{2p_{k}q_{k}}$ | 1 | *F* |
| A8 (*F^I^*) | $\frac{3p_{k}-1}{q_{k}}$ | $\frac{1}{2p_{k}q_{k}}-3$ | $\frac{2-3p_{k}}{p_{k}}$ | *F* |
| A9 (*F^III^*) | $\frac{p_{k}}{q_{k}}$ | –1 | $\frac{q_{k}}{p_{k}}$ | *F* |
| Frequencies | $p_{k}^{2}+p_{k}q_{k}F$ | $2p_{k}q_{k}\left(1-F\right)$ | $q_{k}^{2}+p_{k}q_{k}F$ |  |

**Table A1.** Values of the different estimators of the inbreeding coefficient from the allele frequencies considering the three possible genotypes at a locus. *p_k_* and *q_k_* = 1 – *p_k_* are the frequencies of that allele at the base population and *F* is the expected inbreeding coefficient.

Consider now the correlation between the alleles carried by individuals at a given locus. Cockerham (1969) showed that the expected covariance between the gene frequencies of the alleles of two individuals for a biallelic locus is cov*_M,ij_* = *f_ij_* *p_k_ q_k_*, where *p_k_* and *q_k_* = 1 – *p_k_* are again the base population allele frequencies, so that *f_ij_* = cov*_M,ij_* / (*p_k_ q_k_*) (see Toro et al., 2011). Thus, an estimator of the coancestry between individuals *i* and *j* is then (again for a single locus for simplicity)

$\hat{f}_{ij}=\frac{\mathrm{cov}_{M,ij}}{p_{k}q_{k}}=\frac{\left(p_{ki}-p_{k}\right)\left(p_{kj}-p_{k}\right)}{p_{k}q_{k}}$ , (A5)

where *p_ki_* is the frequency of one of the alleles of the locus in individual *i* (equal to 0, ½ or 1 if the individual carries none, one or two copies of the allele), and *p_k_* is again the average frequency of that allele in the reference population. This is the estimator proposed by Van Raden (2008) for the genomic relationship matrix. Considering the self-coancestry (*f_ii_*),

$\hat{f}_{ii}=\frac{\left(p_{ki}-p_{k}\right)^{2}}{p_{k}q_{k}}$ . (A6)

Applying again Eqn. A1, we obtain

$\hat{F}_{i}=\frac{2\left(p_{ki}-p_{k}\right)^{2}}{p_{k}q_{k}}-1$ , (A7)

and noting that *x_k_* = 2*p_ki_*, it becomes

$\hat{F}_{i}=F^{I}=\frac{\left(x_{k}-2p_{k}\right)^{2}}{2p_{k}q_{k}}-1$ , (A8)

which is Eqn. 1 of the main text (Estimator *F^I^*) for the case of a single locus. The application of Eqn. A8 to the three genotypes is given in the second row of Table A1.

Finally, Yang et al. (2010) proposed an alternative estimator predicted to have lower error variance than the previous ones (Eqn. 3 of the main text; Estimator *F^III^*),

$\hat{F}_{i}=F^{III}=\frac{x_{k}^{2}-\left(1+2p_{k}\right)x_{k}+2p_{k}^{2}}{2p_{k}q_{k}}$ , (A9-a)

Substituting *x_k_* = 2*p_ki_* into Eqn. A9-a,

$\hat{F}_{i}=F^{III}=\frac{\left(p_{ki}-p_{k}\right)^{2}-p_{ki}\left(1-p_{ki}\right)}{p_{k}q_{k}}$ , (A9-b)

which shows the difference with Eqn. A7. The numerator of Eqn. A9-b gives the squared deviation of the allele frequencies of individuals from the population mean minus the expected variance within individuals. The application of Eqn. A9 to the three genotypes is given in the third row of Table A1. Eqn. A9 gives also the correlation between the gametic frequencies of uniting gametes at a locus (Yang et al., 2010), i.e.,

$F^{III}=\frac{\left(p_{kg1}-p_{k}\right)\left(p_{kg2}-p_{k}\right)}{p_{k}q_{k}}$ , (A9-c)

where *p_kg_*_1_ and *p_kg_*_2_ are the frequencies of a given allele in two uniting gametes, being 1 or 0 if the allele is carried by the gamete or not, respectively. For a given individual, *p_ki_* = (*p_kg_*_1_ + *p_kg_*_2_)/2, and substituting this into Eqn. A9-b, we obtain Eqn. A9-c. Applying Eqn. A9 for the three genotypes with values *p_kg_*_1_/*p_kg_*_2_ being 0/0, 0/1 and 1/1, respectively, we obtain again the values in the third row of Table A1.

As mentioned above, the coefficient of inbreeding of an individual (*F_i_*) refers to a reference base population, e.g. an earlier generation in the population. If the marker allele frequencies of the reference population (*p_k_*) are included in Eqns. A4, A8 and A9, as it should be, these are expected to estimate the genealogical (IBD) inbreeding coefficient (*F_i_*). Thus, if the values from Eqns. A4, A8 and A9 for each genotype in Table A1 are each multiplied by the corresponding expected genotypic frequencies (fourth row in Table A1) and added, this results in the inbreeding coefficient *F*, as expected. This would also apply for multiple markers in the absence of linkage disequilibrium and selection. However, the base population allele frequencies (*p_k_*) are almost always unknown and the current frequencies of the population are used instead in the above expressions. In that case, the estimators provide deviations of the observed genotypic frequencies from the current expected frequencies under random mating, with positive values generally denoting an excess of homozygotes and negative values a defect. The different summations over loci produce different outcomes and thus, they are expected to show different correlations with IBD measures of inbreeding (*F*).

**References**

Caballero, A. (2020). *Quantitative Genetics*. Cambridge University Press. Cambridge, U.K.

Crow, J. F. & Kimura, M. (1970). *An Introduction to Population Genetics Theory*. Harper & Row, New York, USA.

Cockerham, C. C. (1969). Variance of gene frequencies. *Evolution*, 23, 72–84.

Li C. C., & Horvitz D. G. (1953). Some methods of estimating the inbreeding coefficient. *American Journal of Human Genetics*, 5, 107–117.

Malécot, G. (1948). *Les Mathématiques de l'Hérédité*. Masson et Cie, Paris, France.

Toro, M. A., García-Cortés, L. A., & Legarra, A. (2011). A note on the rationale for estimating genealogical coancestry from molecular markers. *Genetics Selection Evolution*, 43, 27.

Van Raden, P. M. (2008). Efficient methods to compute genomic predictions. *Journal of dairy Science*, 91, 4414–4423.

Wright, S. (1922). Coefficients of inbreeding and relationship. *American Naturalist*, 56, 330–338.

[Yang](https://www.ncbi.nlm.nih.gov/pubmed/?term=Yang%20J%5BAuthor%5D&cauthor=true&cauthor_uid=20562875), J., [Benyamin](https://www.ncbi.nlm.nih.gov/pubmed/?term=Benyamin%20B%5BAuthor%5D&cauthor=true&cauthor_uid=20562875), B., [McEvoy](https://www.ncbi.nlm.nih.gov/pubmed/?term=McEvoy%20BP%5BAuthor%5D&cauthor=true&cauthor_uid=20562875), B. P., [Gordon](https://www.ncbi.nlm.nih.gov/pubmed/?term=Gordon%20S%5BAuthor%5D&cauthor=true&cauthor_uid=20562875), S., [Henders](https://www.ncbi.nlm.nih.gov/pubmed/?term=Henders%20AK%5BAuthor%5D&cauthor=true&cauthor_uid=20562875), A. K., [Nyholt](https://www.ncbi.nlm.nih.gov/pubmed/?term=Nyholt%20DR%5BAuthor%5D&cauthor=true&cauthor_uid=20562875), D. R., [Madden](https://www.ncbi.nlm.nih.gov/pubmed/?term=Madden%20PA%5BAuthor%5D&cauthor=true&cauthor_uid=20562875), P. A., [Heath](https://www.ncbi.nlm.nih.gov/pubmed/?term=Heath%20AC%5BAuthor%5D&cauthor=true&cauthor_uid=20562875), A. C., [Martin](https://www.ncbi.nlm.nih.gov/pubmed/?term=Martin%20NG%5BAuthor%5D&cauthor=true&cauthor_uid=20562875), N. G.,  [Montgomery](https://www.ncbi.nlm.nih.gov/pubmed/?term=Montgomery%20GW%5BAuthor%5D&cauthor=true&cauthor_uid=20562875), G. W., [Goddard](https://www.ncbi.nlm.nih.gov/pubmed/?term=Goddard%20ME%5BAuthor%5D&cauthor=true&cauthor_uid=20562875), M. E., & Visscher, P. M. (2010). Common SNPs explain a large proportion of heritability for human height. *Nature Genetics*, 42, 565–569.

Zhang, Q., Calus, M. P., Guldbrandtsen, B., Lund, M. S., & Sahana, G. (2015). Estimation of inbreeding using pedigree, 50k SNP chip genotypes and full sequence data in three cattle breeds. *BMC Genetics*, 16, 88.

**Figure S1**. Proportional deviation for each simulation replicate of the estimates of the rate of inbreeding depression (ID) obtained with different measures of the inbreeding coefficient with marker data (*F^III^*, *F_HOM_*, F_ROH-1_ and F_ROH-5_; see text), with respect to the true simulated ID. Simulations assume different population sizes (*N*).

**
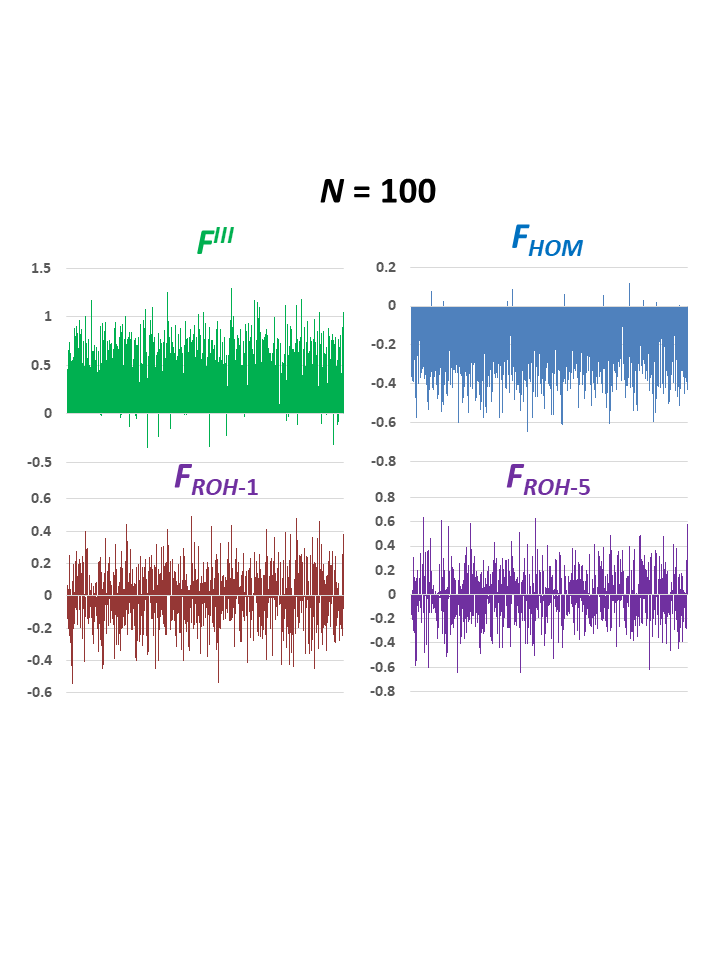
**

**
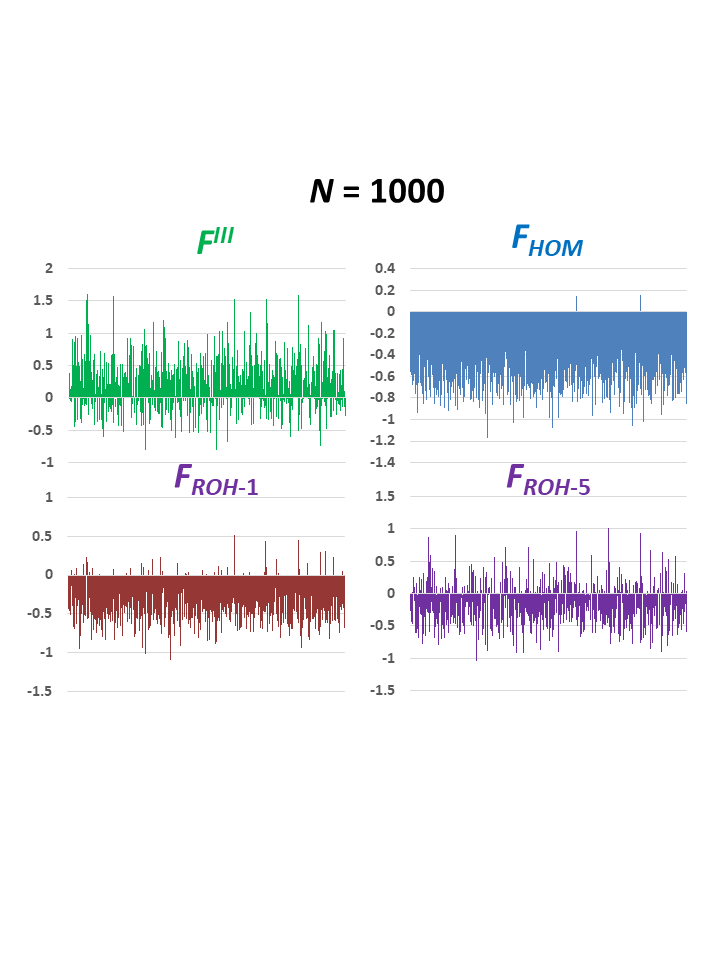

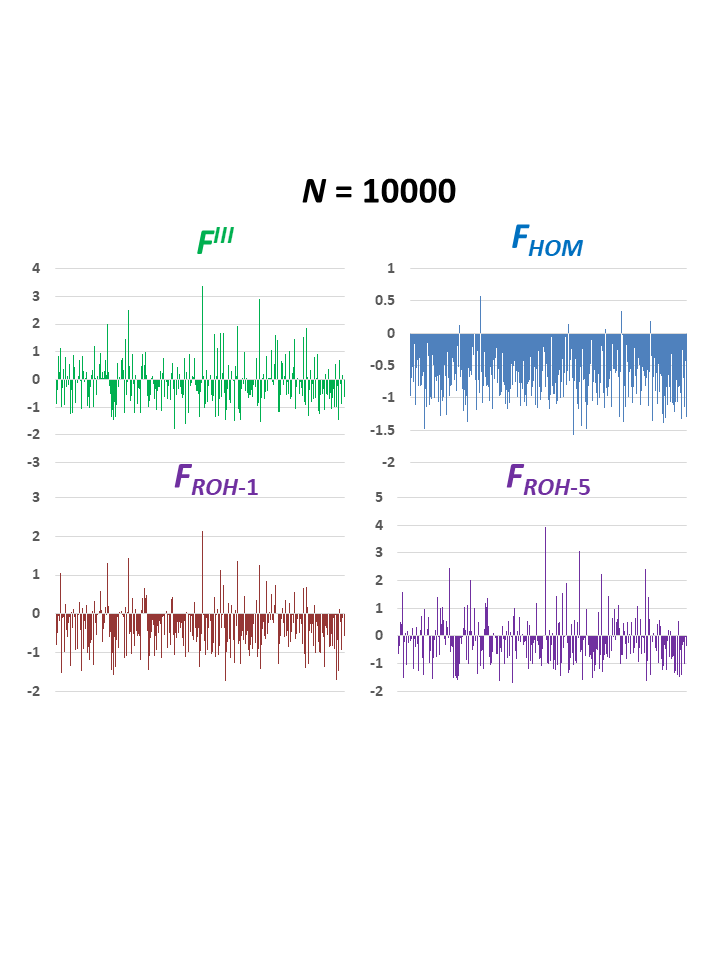
**

**Figure S2**. Proportional deviation of the estimates of the rate of inbreeding depression (ID) obtained with different estimators of the inbreeding coefficient with marker data (see main text), with respect to the true simulated ID. Simulations assume different population sizes (*N*). The dot is the mean deviation and the bar indicates the 95% of the distribution of simulated replicates. The results include all simulated scenarios included in Figures 1 and 3 of the main text.
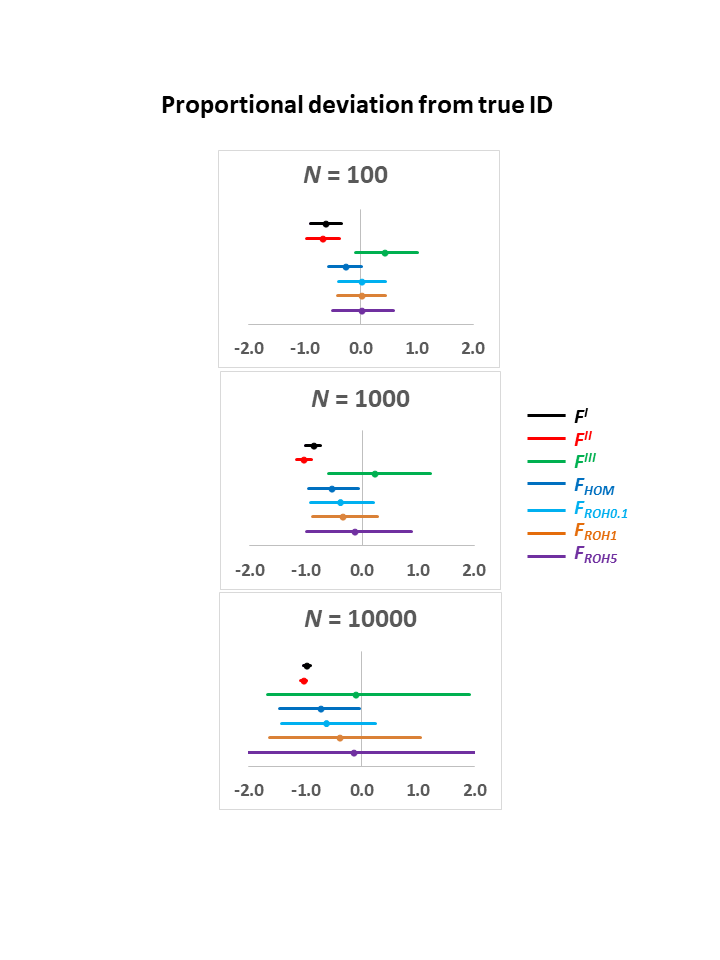


**Figure S3**. Proportional deviation of the estimates of the rate of inbreeding depression (ID) obtained with different estimators of the inbreeding coefficient with marker data (see main text), with respect to the true simulated ID. Simulations assume different population sizes (*N*). The dot is the mean deviation and the bar indicates the 95% of the distribution of simulated replicates. The results refer to simulations carried out with a computer program alternative to SLiM. The number of simulations were 1000, 94 and 66 for *N* = 100, 1000 and 5000, respectively. Each result is obtained as the average of 200 analyses carried out during the last 200 generations of each simulation, hence the short error bars observed.


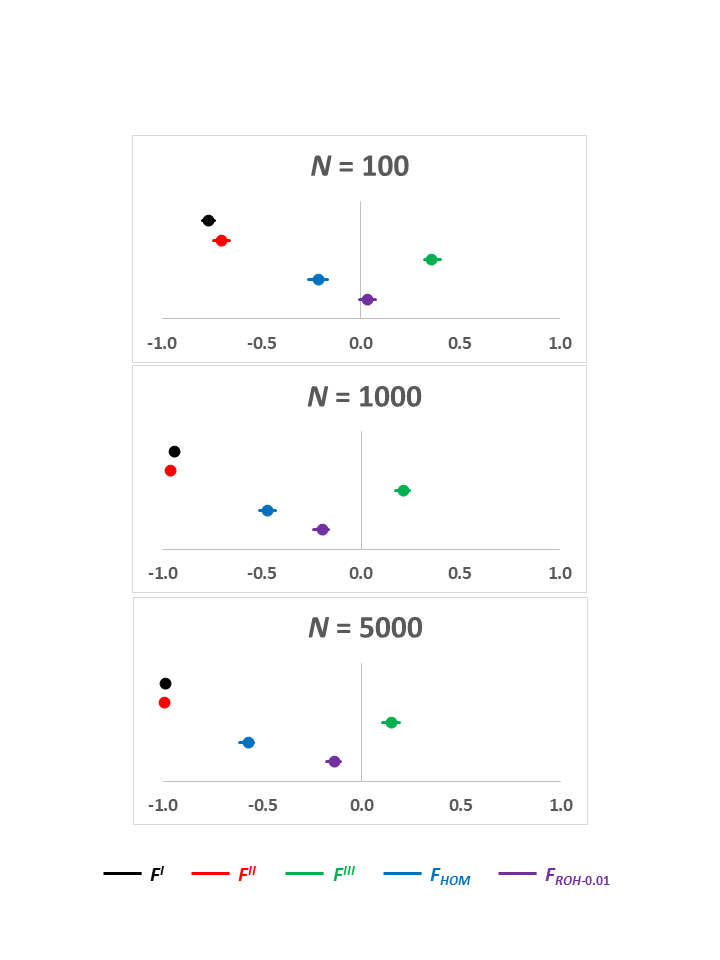


**Figure S4**. Allele frequencies and effects for different population sizes (*N*). (a-c): distribution of minor allele neutral SNP frequencies; (d-f): distribution of minor allele QTL frequencies; (g-i): average homozygous selection coefficient (*s*) against allele frequency; (j-l): contribution to inbreeding depression of QTLs with different allele frequencies.

**
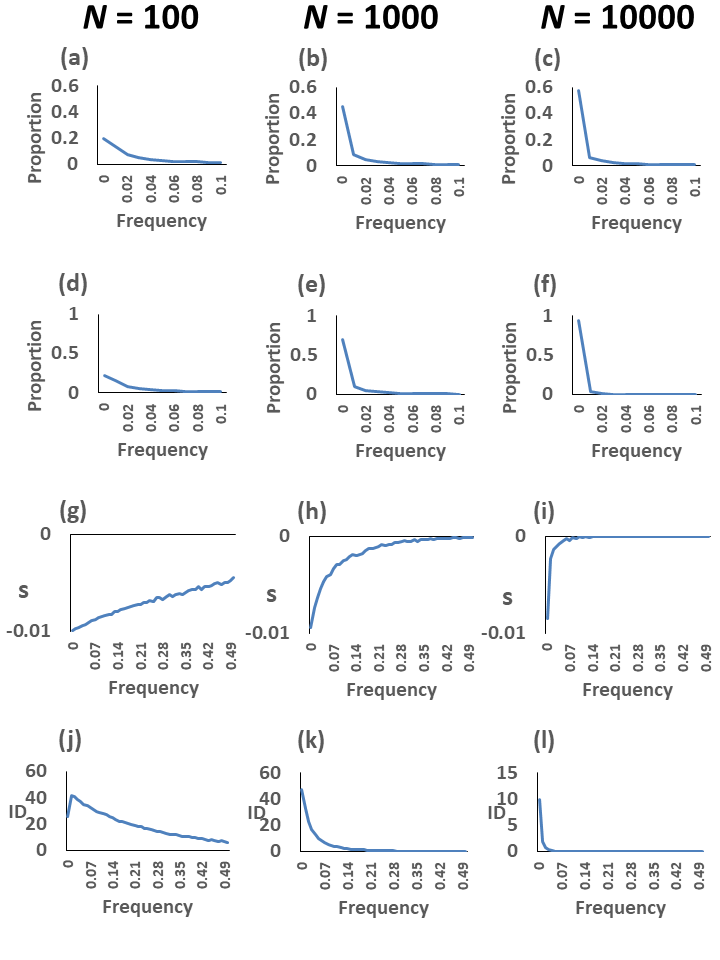
**
